# Evaluating real-world uptake of the online Military to Civilian Adjustment and Reintegration Measure among former military members

**DOI:** 10.1080/00049530.2026.2664166

**Published:** 2026-05-03

**Authors:** Karolina Alichniewicz, Gina Fisher, Angeli Tabinga, Camila Guindalini

**Affiliations:** a Gallipoli Medical Research, Greenslopes Private Hospital, Brisbane, Queensland, Australia; b Faculty of Health and Behavioural Sciences, The University of Queensland, Brisbane, Queensland, Australia

**Keywords:** Military-to-civilian transition, psychosocial adjustment, Australian defence force, reintegration, veterans, implementation science

## Abstract

**Objective:**

This study evaluated real-world engagement with the online Military to Civilian Adjustment and Reintegration Measure (M-CARM) platform, described user characteristics and usage patterns, and examined score distributions, factor structure and variability in a naturalistic online sample relative to the original validation study.

**Method:**

Website analytics (2022–2025) and M-CARM completion data (December 2020–February 2025) were analysed to assess reach, uptake, and engagement. Following data cleaning, 1,101 unique responses were retained. Group differences were examined using t-tests, ANOVAs, chi-square tests, and logistic regression. A confirmatory factor analysis (CFA) evaluated the fit of the hypothesised five-factor structure.

**Results:**

The platform received 10,155 site visits with an 11.2% completion rate among users who accessed the assessment page. Users were predominantly male, aged 26–45 and from the Army. The CFA demonstrated acceptable model fit and strong item loadings. Exploratory analyses identified that Navy users had lower total scores and higher odds of Resentment and Regret, and older veterans (56–75) were less likely to meet criteria for this need compared with younger groups.

**Conclusions:**

This is the first study to examine real-world engagement with the M-CARM platform, highlighting the M-CARM’s translational potential as a scalable, accessible digital tool for identifying reintegration needs among veterans.

## KEY POINTS

**What is already known about this topic**:

(1) Reintegration into civilian life following military separation is a complex and highly individual process influenced by service experiences, personal circumstances, and cultural adjustment.

(2) Although research shows that the years immediately after separation are a time of heightened vulnerability, reintegration challenges can persist well beyond the initial transition period.

(3) Online assessment platforms reduce stigma, improve accessibility, and enable large-scale data to examine user demand and characteristics.

**What this topic adds**:

(1) Demostrates real-world engagement with the M-CARM platform beyond controlled research environments.

(2) Confirms scoring consistency with the original validation study.

(3) Shows robust psychometric performance under naturalistic conditions, reinforcing the tool’s reliability and suitability for applied service contexts.

## Introduction

Reintegration into civilian life following permanent separation from military service is widely conceptualised as a multifaceted and protracted process requiring adjustment across numerous domains (Elnitsky et al., 2017). Transition experiences are highly heterogeneous and are shaped by military exposures, duration and conditions of service, personal circumstances, and the motivations surrounding discharge (Elnitsky et al., 2017; Pedlar et al., 2019). Post-discharge, veterans commonly navigate shifts in family and relational dynamics, employment circumstances, health conditions, and civilian social networks, each representing potential sources of strain during reintegration (Elnitsky et al., 2017). Epidemiological research indicates that the years immediately following separation constitute a period of heightened vulnerability, with 46% of Australian ex-service personnel within 5 years of discharge meeting criteria for a mental health diagnosis (Van Hooff et al., 2018). Notably, reintegration difficulties may endure long after transition. Australian data shows that approximately 50% of veterans reported feeling they had never fully readjusted to civilian life despite being, on average, a decade post-separation (Romaniuk et al., 2020).

Qualitative investigations further underscore the complexity of this process, highlighting experiences of identity discontinuity, cultural dissonance, and loss of community (Kerr et al., 2023; Romaniuk & Kidd, 2018). A recent thematic meta-synthesis of 28 studies delineated six overarching themes characterising military-to-civilian transition: military institutionalisation, military – civilian cultural contrast, the three S’s (Stereotypes, Skills, and Support), losses of identity, reconnection with family and civilian networks, and transition facilitators, illustrating the breadth of cultural and psychosocial challenges involved (Sachdev & Dixit, 2024). These findings align with contemporary conceptualisations positioning psychological and cultural adjustment as central to post-service wellbeing (McCaslin et al., 2021; Romaniuk et al., 2020) and reinforce recent Australian policy recommendations emphasising improved transition support (Royal Commission into Defence and Veteran Suicide (2024).

In response to a need for empirically grounded assessment of reintegration challenges, the Military to Civilian Adjustment and Reintegration Measure (M-CARM) was developed to assess key determinants of successful transition, with particular emphasis on cultural and psychological adjustment (Romaniuk et al., 2020). The instrument comprises five sub-domains representing areas of need related to adjustment and reintegration to civilian life: *Purpose and Connection* (PaC), *Help Seeking* (HS), *Resentment and Regret* (RaR), *Regimentation* (Reg), and *Beliefs about Civilians* (BaC). Developed and validated with Australian ex-serving personnel, the M-CARM has been publicly accessible via an online platform since December 2020 (www.m-carm.org), enabling self-administration and automated scoring for individuals, clinicians, and service providers.

Understanding how such measures are adopted and utilised in real-world contexts is essential for determining their reach, acceptability, and translational impact (Greenhalgh et al., 2017). Online assessment platforms offer distinct advantages, including reduced stigma-related barriers, accessibility for geographically dispersed populations, and the capacity to generate large-scale, routinely collected data (Lieberman, 2012). These data provide opportunities to examine demand for the measure, characteristics of users, and patterns in scoring outside the controlled research environment. Since the M-CARM’s availability as an online tool, its real-world utilisation and the characteristics of those who choose to engage with it have not been examined. Consequently, there is limited understanding about the contemporary patterns of demand, user engagement, or how M-CARM scores are distributed among individuals independently seeking support for their transition and reintegration. Understanding these patterns is essential for optimising the reach, acceptability, and sustainability of evidence-based digital resources in the veteran community.

Further, this gap is situated within a broader evidence base that has predominantly focused on global indicators of post-discharge mental health rather than domain-specific psychosocial adjustment. Consistent predictors of poorer outcomes include unplanned discharge and younger age at separation (Buckman et al., 2013; Burdett et al., 2021), yet little is known about how reintegration needs manifest among users engaging with self-guided tools in everyday contexts.

Guided by the *Reach* and *Adoption* dimensions of the RE-AIM framework (Glasgow et al., 1999), and complemented by psychometric analyses, the present study investigates the real-world utilisation of the M-CARM online measure. Using routinely collected naturalistic data, we aimed to: i. characterise demand and user engagement with the tool over time; ii. describe user characteristics and usage patterns among individuals accessing the platform; iii.examine the distribution of M-CARM scores in real-world conditions, and the extent to which these patterns align with those reported in the original validation study; iv. evaluate whether the M-CARM’s five-factor structure replicates in a naturalistic online sample using confirmatory factor analysis (CFA); and v. provide exploratory insights into score variability across available demographic and service characteristics.

By focusing on real-world engagement and user M-CARM profiles, this study offers the first evidence of how a validated measure of military cultural and psychological adjustment to civilian life is accessed and utilised outside controlled research settings.

## Materials and methods

### Data collection

The M-CARM website (www.m-carm.org) was launched in December 2020. This report includes data collected from the time of launch to 14 February 2025. Data collected as part of the M-CARM platform included item responses, user email addresses, and basic demographic data. Google analytics data was available from September 2022 to 14 February 2025 including sources of web-page traffic, website visits, and page views.

### Ethics

The Department of Defence and Veterans’ Affairs Human Research Ethics Committee has deemed this study to be a quality assurance activity that upholds the principles of the National Statement on Ethical Conduct in Human Research and the Ethical Consideration in Quality Assurance and Evaluation Activities (Project ID: 2023/BN65721572). In the privacy policy statement that participants have access to when deciding to complete M-CARM online, they are informed about the organisation’s commitment to comply with the Privacy Act 1988 (Cth) (Privacy Act), including the Australian Privacy Principles.

### Measures

#### User characteristics

Demographic and service questions were integrated into the M-CARM platform in June 2021. Participants were asked to indicate their age range, service background (Navy, Army, Air Force; selected all that applied), and gender (male, female). Each questions included the option *“Prefer Not to Answer”*.

#### Military to Civilian Adjustment and Reintegration Measure (M-CARM)

The M-CARM is a 21-item questionnaire that measures factors important for reintegrating into civilian life following permanent separation from military service. Factors include:

- Purpose and Connection (e.g., “I have things that give me a sense of purpose, outside of paid employment”);
- Help Seeking (e.g., “I know how to access professional support for my mental health”);
- Resentment and Regret (e.g., “The military broke me and then kicked me out”);
- Regimentation (e.g., “some of my military habits cause problems for me”); and
- Beliefs about Civilians (e.g., “civilians are disrespectful and rude”).

Items are rated on a Likert scale from 1 (“*Strongly Disagree*”) to 5 (“*Strongly Agree*”). Total scores range from 21 to 105, with higher scores indicating better psychological adjustment and cultural reintegration. To enable detection of key areas of need, a scoring profile can be created by calculating an average score for each sub-domain. Sub-domain average scores range from 1 to 5, with a score of 3 or below indicating this may be an “area of need” in which a respondent could benefit from targeted support.

### Data analysis

At the time of analysis, a total of 2,115 completed questionnaires had been recorded on the M-CARM website. M-CARM case data was checked and cleaned to maximise confidence in the validity of responses. Cases were removed if associated with an email address indicating it was a test response (*n* = 435). Cases were removed if the pattern of responses was unlikely to resemble a valid response pattern (e.g., selecting *“Strongly Agree”, “Neither Agree or Disagree” or “Strongly Disagree”* for every question; *n* = 29). To ensure that each email address corresponded to a unique user, cases were removed if there were inconsistencies in key demographic variables (e.g., cases with the same email address had differing gender and service type; *n* = 456). If an email address was associated with more than one M-CARM response, all subsequent responses were removed (accounting for *n* = 94 completions). Finally, cases were removed if they were flagged by two or more indicators of careless responding, including longstring, intra-individual response variability (IRV), and Mahalanobis distance, identified using the R package “careless” (*n* = 3; Yentes & Wilhelm, 2023). Following case removal, the final dataset included *N* = 1,098 cases.

Descriptive statistics were computed to summarise the sources of website traffic and the geographic location (based on IP address) of visitors to the M-CARM platform, collected from February 2022 to February 2025. The “Health Practitioners” page was only added in April 2023, and as such, metrics relating to this section of the platform were collected from April 2023 onwards. Two groups were considered: all site visitors (anyone who accessed the platform) and M-CARM users (those who completed the measure and provided demographic information). Data from M-CARM completions were then analysed to explore whether total scores and domain-specific areas of need differed according to age, gender, and service background.

Independent samples *t*-tests were conducted to compare mean total M-CARM scores by gender, while one-way analyses of variance (ANOVAs) were used to assess differences across age categories and service branches. Where significant group effects were identified, Tukey post hoc tests were applied to determine pairwise differences. To explore associations between demographic variables and the proportion of users meeting criteria for each area of need, chi-square tests of independence were performed. To further examine these patterns and account for potential confounding variables, a binary logistic regression analysis was conducted following the initial univariate comparisons. All analyses were conducted using R, with significance set at *p* < .05.

A confirmatory factor analysis (CFA) using structural equation modelling with maximum likelihood estimation was conducted on the online M-CARM user sample to test whether the factor structure reported by Romaniuk et al. (2020) replicated in this dataset. Analyses were performed using the lavaan package in R. Consistent with the original study, model fit was evaluated using the Chi-square statistic (χ^2^), Root Mean Square Error of Approximation (RMSEA), Comparative Fit Index (CFI), Tucker – Lewis Index (TLI), and Normed Fit Index (NFI). These indices were examined alongside the χ^2^ test because χ^2^ is highly sensitive to minor deviations from perfect fit in large samples (Stevens, 2012). Excellent model fit is typically indicated by CFI and TLI values ≥ .95 (Bentler, 1990), RMSEA ≤ .05 (Browne & Cudeck, 1993), and NFI ≥ .90 (Bentler, 1990). In line with common practice, acceptable model fit was defined as CFI and TLI ≥ .90 and RMSEA ≤ .08 (Hu & Bentler, 1999).

## Results

### M-CARM online reach and uptake

Google Analytics revealed that there were 10,155 sessions on the M-CARM website between February 2022 and February 2025. Users predominantly reached the site through direct channels (i.e., directly via the web URL), accounting for 52% of site visits. 31% of users reached the site via organic channels (i.e., through search engine results), and 15% reached the site via referral traffic (i.e., reaching the site via another site), with the remaining proportion being organic social (unpaid links shared on social media), or other traffic pathways. 58% of those who accessed the M-CARM platform were from IP addresses in Australia, with the next highest proportion coming from the U.S. (28.2%); U.K. (2.7%); Canada (1.8%); and India (1.8%). The remaining percentage of responses were linked to IP addresses from various other countries (7.5 %). The “Health Practitioners” page, established in April 2023 to provide information about the M-CARM for clinical use, recorded 1,336 views between its launch and February 2025.

Because website analytics were made available for the website that hosts M-CARM at a later date, precise calculation of conversion rates across the entire data collection period was limited. Between July 2022 and February 2025, the “Take the M-CARM” page was viewed 4,752 times. Based on 533 valid M-CARM completions within the same timeframe, approximately 6% of those who visited the website completed the M-CARM, and 11.2% of those who viewed the “Take the M-CARM” page completed the M-CARM. Notably, 7.8% of M-CARM completions were associated with organisational or health-service email addresses, suggesting that the measure was potentially administered by practitioners as part of service delivery by a practitioner (*n* = 86).

### M-CARM users demographic and service characteristics

Demographic questions were completed by 99.0% of M-CARM users (*n* = 1,087). Table 1 provides an overview of user characteristics. Participants represented a broad demographic range, with the majority aged between 26 and 55 years (75%), and most identifying as male (74.9%). The most reported service background was Army (63.7%), followed by Navy (18.2%) and Air Force (17.1%). Given the small proportion of responses selecting the *“Prefer Not to Answer”* option for demographic variables, these entries were considered missing data from the corresponding analyses to maintain interpretability.

**Table 1.** Demographic and characteristics for the M-CARM users sample.

Variable	%	*n*
Age category		
18–25	8.90%	96
26–35	27.00%	292
36–45	25.00%	271
46–55	23.20%	251
56–65	13.10%	142
66–75	2.10%	23
76 and over	0.60%	7
Gender		
Female	25.10%	271
Male	74.90%	808
Service Type		
Navy	18.20%	180
Army	63.70%	631
Air Force	17.10%	169
More than one	1.10%	11

### M-CARM total score and areas of need

#### Total sample

The mean M-CARM total score for the full sample was 64.42 (*SD* = 17.31; range = 21–105). In addition, across the full sample, *Regimentation* represented the most identified area of need, with 68.31% of users meeting the threshold (*n* = 750). This was followed closely by *Beliefs about Civilians* (67.76%, *n* = 744) and *Resentment and Regret* (59.93%, *n* = 658). *Purpose and Connection* was identified in 45.81% of users (*n* = 503), whereas *Help Seeking* was the least frequently endorsed domain, with 26.50% meeting the threshold (*n* = 291). Overall, more than half of the sample (55.59%, *n* = 611) met criteria for three to five areas of need. Notably, 78 (7.10%) users have returned to complete M-CARM repeatedly in the time of this evaluation. To explore whether users who completed the M-CARM more than once differed from single-time users in their needs profile, total and subscale scores were compared between the two groups. No statistically significant differences were observed.

#### Comparison of online sample and original validation cohort

Table 2 depicts comparison of the M-CARM domain scores from online users with the original validation study participants. Participants responded to 21 items using a 5-point Likert scale. Thirteen items were reverse-scored prior to analysis (Romaniuk et al., 2020). Domain scores were calculated by averaging item responses corresponding to each domain. Largely, the total and sub-domain scores are similar, suggesting comparable levels of adjustment and reintegration across cohorts.

**Table 2.** Comparison of summed M-CARM domain scores between online users and original validation cohort.

	Descriptives (Validation Cohort^a^)			Descriptives (M-CARM online users)		
Factor	*Sum*	*SD*	Range	*Sum*	*SD*	Range
Purpose and connection	19.71	6.95	6–30	19.06	6.82	6–30
Help seeking	15.18	3.79	4–20	14.80	3.59	4–20
Beliefs about civilians	9.21	4.08	4–20	10.88	4.08	4–20
Resentment and regret	9.13	3.95	3–15	8.68	3.83	3–15
Regimentation	10.9	4.08	4–20	10.98	3.96	4–20
Total	64.13	16.84	26–105	64.43	17.29	21–105

^a^Data are from Romaniuk et al. (2020).

Note: Scores represent the sum of item responses within each of the five M-CARM domains. All 21 M-CARM items were rated on a 5-point Likert scale; 13 items were reverse-scored.

A confirmatory factor analysis (CFA) was conducted to examine the hypothesised 5-factor structure of the M-CARM. A single-factor model was also estimated to enable comparison of model fit indices. The five-factor model demonstrated acceptable fit, χ^2^ (179) = 936.83, RMSEA = 0.062, CFI = 0.925, TLI = 0.912. All items loaded significantly on their intended factors (standardised loadings range = 0.482–0.846), with the majority above 0.60, indicating good indicator reliability. The latent factors were positively correlated, supporting the theoretical relationships among constructs. R^2^ values indicated that most items had moderate to high variance explained by their respective factors (range = 0.232–0.716). The alternative single-factor model showed poorer fit, χ^2^ (189) = 2936.50, RMSEA = 0.115, CFI = 0.728, TLI = 0.698. Overall, the CFA provides support for the 5-factor structure of the M-CARM and the validity of its subscales in a real-world online sample.

### Group differences in M-CARM scores and needs

The following section presents findings from the analysis of M-CARM data, focusing on two key outcome variables:

1. total M-CARM score, and
2. the proportion of users meeting criteria for each individual area of need.

Analyses examined variation in these outcomes across gender, age group, and service type, with higher scores indicating better psychological adjustment and cultural reintegration. The distribution of total M-CARM scores was visualised using histograms, which can be found in the Supplementary Material.

#### Gender differences

Male users reported slightly higher M-CARM total scores (*M* = 64.97 ± 16.95) than female users (*M* = 62.61 ± 18.29). However, this difference did not reach statistical significance (*t* (436) = −1.88, *p* = 0.061, d = 0.137).

The proportion of male and female users meeting individual areas of need was similar for both genders, in relation to *Purpose and Connection, Beliefs about Civilians*, and *Regimentation*, as presented in Table 2. In contrast, a significantly higher proportion of female users met criteria for *Help Seeking* (*X*^2^ (1) = 4.41, *p* = 0.036. *Phi* = 0.064) and *Resentment and Regret* needs *(X*^2^ (1) = 9.501, *p* = 0.002, *Phi* = 0.094), with small effect sizes.

#### Service type differences

Total M-CARM scores were compared across service type. Mean scores were as follows: Navy (*M* = 61.77 ± 16.13), Army (*M* = 65.33 ± 17.23), Air Force (*M* = 67.78 ± 17.02), and More than one (*M* = 58.46 ± 9.88). There was a significant effect of service background on M-CARM total scores, *F*(3, 987) = 4.42, *p* = 0.004, with a small effect size (*η^2^* = 0.01). Post-hoc comparisons indicated that Navy participants scored significantly lower than Air Force participants (95% CI [1.41, 10.75], *p* = 0.005). There was no significant difference between Navy and Army participants (95% CI [−0.04, 7.33], *p* = 0.054), and Air Force and Army participants (95% CI [−1.34, 6.22], *p* = 0.35). Further, no significant differences between the “more than one” group and Navy, Army or Airforce were found (*p*’s > 0.05). After controlling for age and gender, the overall effect of service type remained significant *F*(3, 973) = 4.63, *p* = 0.003. Tukey-adjusted comparisons of the estimated marginal means showed that Navy participants scored lower than Army participants (adjusted mean difference = −3.76, *p* = 0.049, Cohen’s d = −0.22) and Air Force participants (adjusted mean difference = −6.14, *p* = 0.005, Cohen’s d = −0.36). No significant differences were found between More than one and Army participants (adjusted mean difference = −7.74, *p* = 0.48, Cohen’s d = −0.46), More than one and Airforce participants (adjusted mean difference = −10.12, *p* = 0.26, Cohen’s d = −0.60), Navy and More than one participants (adjusted mean difference = 3.98, *p* = 0.89, Cohen’s d = 0.23), and Army and Air Force participants (adjusted mean difference = −2.38, *p* = 0.39, Cohen’s d = −0.14).

The proportion of M-CARM users scoring below the threshold for each sub-domains was also examined by service type (Table 3). Overall, patterns of need were broadly similar across groups. However, differences in the proportion of users meeting criteria for the *Beliefs about Civilians (*X^2^(3) = 8.43, *p* = 0.038, *Cramer’s V* = 0.092), *Regimentation* (X^2^(3) = 8.77, *p* = 0.032, *Cramer’s V* = 0.094), and *Resentment and Regret* (X^2^(3) = 13.09, *p* = 0.004, *Cramer’s V* = 0.115) domains differed significantly across service backgrounds. Notably, users with a Navy service background showed higher proportions meeting criteria for these areas of need, compared with those from Army or Air Force, as shown in Table 3.

**Table 3.** Proportion (%) and number (n) of users meeting each M-CARM area of need by total sample, gender, service type, and age group.

Subgroup	Purpose and connection	Beliefs about civilians	Help seeking	Regiment-ation	Resentment and regret
Total sample	45.8 (503)	67.7 (744)	26.5 (291)	68.3 (750)	59.9 (658)
Gender					
Male	45.4 (367)	68.2 (551)	24.6 (199)	68.3 (552)	57.4 (464)
Female	48.0 (130)	66.4 (180)	31.4 (85)	69.0 (187)	68.3 (185)
Service type					
Navy	50.0 (90)	68.9 (124)	22.8 (41)	74.4 (134)	71.1 (128)
Army	44.4 (280)	67.0 (423)	25.5 (161)	68.0 (429)	56.4 (356)
Air Force	43.2 (73)	60.9 (103)	23.7 (40)	59.8 (101)	56.8 (96)
More than one	54.5 (6)	100.0 (11)	27.3 (3)	72.7 (8)	63.6 (7)
Age group					
18–25	43.8 (42)	62.5 (60)	26.0 (25)	59.4 (57)	65.6 (63)
26–35	50.7 (148)	65.1 (190)	24.0 (70)	73.3 (214)	67.5 (197)
36–45	42.1 (114)	68.3 (185)	27.0 (70)	69.7 (189)	60.1 (163)
46–55	50.2 (126)	71.7 (180)	28.7 (72)	65.7 (165)	59.0 (148)
56–75	41.2 (68)	68.5 (113)	26.7 (44)	67.3 (111)	47.3 (78)

#### Age differences

M-CARM scores and needs proportions were compared across age groups. The number of participants in age groups 56–65, 66–75 and 76+ were: 142, 23, and 7, respectively. The 76+ age category was removed and the age categories 56–65 and 66–75 were grouped to enable simplicity in discussing findings. Participants were distributed as follows: 18–25 years (*n* = 96), 26–35 years (*n* = 292), 36–45 years (*n* = 271), 46–55 years (*n* = 251), and 56–75 (*n* = 165). Mean scores were relatively consistent, ranging from 63.19 to 65.88. Specifically, participants aged 18–25 had a mean score of 65.88 (*SD* = 18.76), those aged 26–35 scored 63.19 (*SD* = 16.30), 36–45 scored 64.58 (*SD* = 17.52), 46–55 scored 63.86 (*SD* = 16.68), and the 56–75 group scored 65.86 (*SD* = 18.86). No statistical significant differences in scores across groups were identified *F*(4, 1070) = 0.89, *p* = 0.47.

The proportion of users meeting different areas of need on the M-CARM were also examined by age groups, with results summarised in Table 3. Overall, patterns were broadly similar across groups; however, a significant difference was observed in the *Resentment and Regret* domain. A higher proportion of 26–35-year-olds met the threshold for this area of need, while the lowest proportion was observed among users aged 56–75 (*X*^*2*^(4) = 19.30, *p* = 0.001, *Cramer’s V* = 0.13).

#### Regression analyses

Following initial comparisons by gender, age and service type, logistic regression models were developed for each M-CARM area of need, as dependent variables, to estimate the unique contribution of the demographic and service type variables while controlling for the potential confounding effects of the others.

After adjusting for service type and age, gender differences in meeting the *Resentment and Regret* and *Help Seeking* needs were no longer statistically significant (RaR: OR = 0.80, 95% CI [0.57, 1.10], *p* = 0.172; HS: OR = 0.87, 95% CI [0.61, 1.25], *p* = 0.452). Similarly, after controlling for age and gender, the differences in *Resentment and Regret* need between Army and Air Force, were no longer significant (*p*’s > 0.05). In contrast, users who served in the Navy had significantly higher odds of meeting the *Resentment and Regret* need compared to those who served in the Army (OR = 1.99, 95% CI [1.38, 2.90], *p* = .0003).

In relation to age group comparisons, the multivariable regression analyses controlling for potential cofounders confirmed the findings observed in the univariate analyses: no significant associations were detected for Help *Seeking*, *Purpose and Connection*, or *Beliefs about Civilians*. However, compared with the youngest age group, veterans aged 56–75 had significantly lower odds of reporting the need *Resentment and Regret*, independent of gender and service type (OR = 0.42, 95% CI [0.24, 0.73], *p* = .002).

## Discussion

This study aimed to evaluate real-world data from the M-CARM online measure to better understand the tool’s reach, uptake, and user characteristics, and to explore how the measure performs outside controlled research conditions. Specifically, it examined patterns of user engagement, the demographic and service-related profile of users, the most common areas of reintegration need, and how these patterns compare to those reported in the original validation study (Romaniuk et al., 2020). Comparative analysis were also conducted to look at score variability across available demographic and service characteristics to generate insights among self-selected veterans seeking support for their adjustment and reintegration into civilian life.

### M-CARM reach, uptake and user engagement

The online M-CARM measures’ usage data, as revealed by Google Analytics, indicates a relevant level of engagement, with 10,155 site accesses over the 36-month period. The conversion rate from site visits to measure completions was 6%, and the completion rate among those who viewed the “Take the M-CARM” page was 11.2%. While these figures reflect promising reach, they also highlight opportunities to optimise user engagement by retaining and converting casual visitors into active users. Together, these patterns mirror broader trends in digital health interventions and illustrate the ongoing challenge of translating initial user interest into consistent, meaningful participation, an issue central to digital health uptake and sustainability (Livieri et al., 2025; Mair et al., 2025). Additionally, a high proportion of direct access suggests strong user intent and possible direct referrals or repeat usage, which could reflect a sound reputation and endorsement within relevant communities.

The geographic distribution of the M-CARM viewers, predominantly from Australia, aligns with the platform’s original target audience but also shows potential for broader international reach. The 28.2% of traffic from the U.S. highlights the potential need for a U.S.-specific, cross-culturally validated version of the measure, which will ensure that variations in language usage, and culture-specific constructs are systematically assessed and appropriately addressed (Zhao et al., 2024). Nevertheless, these findings will help inform ongoing refinement of the M-CARM website, including consideration of collecting additional demographic information, such as country of service or residence and ethnicity, during survey completion to support future cross-cultural validation and more context-sensitive interpretation of responses as the platform expands internationally.

Further, it is important to note that 7.8% of completions were linked to known Australian organisational email addresses, suggesting growing interest from health practitioners in utilising M-CARM for clinical or supportive purposes. These findings support prior work indicating that online questionnaires can be a suitable alternative to more traditional paper-based measures (Riva et al., 2003), while enhancing accessibility and scalability. Zimmerman (2024) further highlights that because self-administered tools rely heavily on respondents’ understanding of language and context, their interpretability depends on careful validation and attention to cultural and linguistic differences, an important consideration for online platforms with international reach (Zimmerman, 2024).

The participant characteristic analysis reveals a user base primarily composed of males (74.4%), majority from the 26–45 years old (51.9%) and from the Army (57.9%), which together account for 51.9% of the total sample. These findings reflect broader patterns in the Australian veteran population and are consistent with other digitally delivered veteran programs such as VetChange, where participants mean age was 31.6 years and 86.9% identified as male (Enggasser et al., 2021). It is of note, however, that a quarter of measure completions were by female veterans which may be seen as a disproportionately high figure, considering that females constitute 13.4% of the veteran population (Australian Bureau of Statistics, 2021). One possible explanation relates to the context and nature of the M-CARM: an anonymous, self-guided digital tool. Such tools may be particularly appealing to female veterans who often navigate unique transition-related challenges and face additional barriers to engaging with traditional support pathways, such as work and caregiving responsibilities or limited availability of gender-appropriate services (Runnals et al., 2014). This suggests that digital platforms may serve as a particularly accessible modality for female veterans and should be considered a promising avenue for delivering relevant and tailored support.

### M-CARM outcomes and user characteristics

The mean and standard deviation of the M-CARM total and sub-domains scores in this study closely matched those reported in the original publication (Romaniuk et al., 2020). This alignment in an independent, naturalistic dataset suggests that the tool is reaching its intended audience and reinforces confidence in its utility to capture key psychosocial challenges associated with the military-to-civilian transition, even when self-administered online. The confirmatory factor analysis further supports this conclusion, demonstrating that the hypothesised five-factor structure showed acceptable model fit, with strong item loadings and positive correlations among latent factors. Together, these findings indicate that both the structure and performance of the M-CARM remain robust when used in real-world conditions, strengthening evidence for the validity and reliability of the measure beyond the original validation setting.

Patterns in M-CARM scoring and areas of need were also compared between users based on gender, age and service background, revealing differences within the different sub-groups. Although factors such as self-selected participation and the inability to verify user identity or collect detailed medical and service characteristics limit the interpretation of these findings, exploratory regression analyses nonetheless suggested relevant patterns. Specifically, users who served in the Navy had significantly lower M-CARM scores compared to Army and Air Force, and higher odds of meeting the *Resentment and Regret* need compared to those who served in the Army, independent of age and gender. This may suggest branch-specific variations in stressors or cultural factors that impact the transition process. Ravindran et al. (2020) emphasise the unique differences in the culture, training experiences, deployment experiences, and demographic compositions between different branches in the military and the need to consider service branch when reporting challenges experienced by veterans. Consistent with this, Brooks and Greenberg (2022) reviewed 63 studies and identified multiple risk factors for poor mental health among seafarers, was associated with a combination of individual vulnerabilities, demanding working conditions, adverse physical environments, and social and organisational stressors – highlighting factors associated with naval service. At the same time, some exposures that may affect health and well-being – such as noise, vibration, fuels or solvents, and shift-related disruption – have been described across a range of military occupational settings rather than being confined to a single service branch (Geretto et al., 2021). For these reasons, future research and support initiatives may benefit from considering both branch-specific contexts and shared occupational stressors in order to more effectively address the challenges veterans face during the transition to civilian life. In addition, incorporating occupational categories alongside service branch, as part of the survey completion, may help future studies better capture these shared experiences and its impact on transition outcomes.

Compared to the youngest age group, veterans aged 56–75 had significantly lower odds of having the need *Resentment and Regret* independent of gender and service type. Interestingly, a study investigating rates of difficult discharge from the military in the U.S. has reported that prevalence rates of difficult discharge were higher than that of the overall population for veterans who were released mid-career (in their 30’s or with 2–19 years of service), medically released veterans, those released from the Army, veterans who deployed multiple times during their career, and junior non-commissioned members (MacLean et al., 2014). Further, Carra et al. (2023) found that participants in the age category 30–49 years reported a more difficult adjustment than their younger and older counterparts. As noted, the naturalist data collection approach employed in this study limits the interpretation of these differences observed. However, the patterns provide valuable insights for future research into the distinct challenges experienced by different veteran subgroups, which can help inform the design of more tailored and effective services and interventions.

### Implications for clinical practice and future research

The current findings emphasise the value of monitoring and understanding how evidence-based tools are used and disseminated beyond controlled research environments. Closing the research implementation gap and translating scientific findings into practical tools and interventions remains a persistent challenge across mental, physical and social health fields (McGinty et al., 2024). A deeper understanding of user engagement patterns can offer valuable insights into strategies for increasing not only reach of evidence-based resources, but also their adoption and long-term sustainability in real world setting (Livieri et al., 2025; Mair et al., 2025).

Our results suggest that the M-CARM’s online delivery model shows promise in bridging this research-to-practice divide. Its accessibility, practicality with automated scoring, and potential for integration into clinical workflows offer scalable opportunities for supporting veterans’ service provision. By using the digital version of the measure, practitioners can collect real-time data, identify adjustment difficulties early, and tailor support services more responsively. Its online format also allows for broader reach across geographic locations, enabling more consistent monitoring and evaluation of veteran wellbeing over time, while reducing administrative burden and improving user engagement. Future research should focus on identifying drivers of engagement and evaluating strategies to increase completion rates, particularly among first-time visitors. Importantly, the initial interest identified by practitioners in this study highlight the need to conduct further studies to understand how to best embed the tool into clinical workflows and service pathways. Also, the development of cross-cultural adaptations of M-CARM, such as a U.S.-specific version, may further expand its utility in other contexts that might benefit from evidence-based tools specifically designed for veteran populations. Finally, the trends observed in our study in relation to potential differences in psychosocial challenges across different age groups and service types emphasise the importance of considering demographic and service-specific factors not only in future transition-related studies, but also when developing support and intervention programs for military personnel and veterans. The findings underscore the importance of offering an online version of the M-CARM, serving as a practical, accessible, and resourceful way to assess challenges veterans face during their transition to civilian life.

### Limitations

The study presents a number of limitations. The voluntary and anonymous nature of the M-CARM completion limits the ability to rely on the information provided or ensure representativeness and likely introduces self-selection bias. Website analytics were partially available since the platform launch limiting the analysis of conversion rates. Additionally, the absence of detailed medical and service-related data further constrains the interpretation of the findings, and, for this reason, results should be understood as indicative trends that inform future work in the field. Another limitation is that, although website analytics indicated that M-CARM website visitors included individuals from multiple countries (such as the US and the UK), we did not capture participants’ country of residence during survey completion. Since international evidence suggests that veteran support systems and reintegration experiences can vary across countries (Carrington, 2023), the lack of this information may have influenced how broadly the findings can be interpreted. Nevertheless, the naturalistic approach of the study, as well as the alignment between real-world data and the original validation findings, suggest that the tool is reaching its intended audience and reinforce its utility and potential to support veterans transitioning to civilian life.

## Conclusion

This is the first study to evaluate how veterans engage with the M-CARM in a real-world setting. Usage data indicates a significant level of engagement, however continued refinement and targeted implementation efforts are needed to strengthen its adoption and potential integration into clinical practice. Although limitations exist in the current dataset, the results suggest that the tool is reaching its intended audience and producing consistent results. The findings highlight the M-CARM’s translational potential and value as a scalable, accessible tool for identifying reintegration needs among veterans. Future research should focus on developing cross-culturally validated versions of the M-CARM, particularly in the U.S., where platform analytics indicate strong user interest, and exploring subgroup-specific reintegration needs to further refine and improve support for transitioning veterans.

## Supplementary Material

Supplemental material

## Data Availability

Data sharing is not available for this article due to the sensitive nature of the research. The script used for data analysis is available from the corresponding author upon reasonable request.

## References

[cit0001] Australian Bureau of Statistics. (2021). *Service with the Australian Defence Force: Census*. ABS. https://www.abs.gov.au/statistics/people/people-and-communities/service-australian-defence-force-census/2021

[cit0002] Bentler, P. M. (1990). Comparative fit indexes in structural models. *Psychological Bulletin*, 107(2), 238–13. 10.1037/0033-2909.107.2.238 2320703

[cit0003] Brooks, S. K., & Greenberg, N. (2022). Mental health and psychological wellbeing of maritime personnel: A systematic review. *BMC Psychology*, 10(1), 139. 10.1186/s40359-022-00850-4 35637491 PMC9150387

[cit0004] Browne, M. W., & Cudeck, R. (1993). Alternative ways of assessing model fit. In K. A. Bollen & J. S. Long (Eds.), *Testing structural equation models* (pp. 136–162). Sage.

[cit0005] Buckman, J. E. J., Forbes, H. J., Clayton, T., Jones, M., Jones, N., Greenberg, N., Sundin, J., Hull, L., Wessely, S., & Fear, N. T. (2013). Early service leavers: A study of the factors associated with premature separation from the UK Armed Forces and the mental health of those that leave early. *European Journal of Public Health*, 23(3), 410–415. 10.1093/eurpub/cks042 22539627

[cit0006] Burdett, H., Fear, N., Wessely, S., & Rona, R. (2021). Military and demographic predictors of mental ill-health and socioeconomic hardship among UK veterans. *BMC Psychiatry*, 21(1), 1–11. 10.1186/s12888-021-03296-x 34225685 PMC8259380

[cit0007] Carra, K., Curtin, M., Fortune, T., & Gordon, B. (2023). Service and demographic factors, health, trauma exposure, and participation are associated with adjustment for former Australian Defense Force members. *Military Psychology*, 35(5), 480–492. 10.1080/08995605.2022.2120312 37615555 PMC10453966

[cit0008] Carrington, J. G. (2023). Review of international practice in the reintegration of veterans: Considerations for Ukraine in the war and post-war context. *United Nations Development Programme (UNDP) in Ukraine*. https://www.undp.org/ukraine/publications/review-international-practice-reintegration-veterans-considerations-ukraine-war-and-post-war-context

[cit0009] Elnitsky, C. A., Fisher, M. P., & Blevins, C. L. (2017). Military service member and veteran reintegration: A conceptual analysis, unified definition, and key domains. *Frontiers in Psychology*, 8(369). 10.3389/fpsyg.2017.00369 PMC5348503 28352240

[cit0010] Enggasser, J. L., Livingston, N. A., Ameral, V., Brief, D. J., Rubin, A., Helmuth, E., Roy, M., Solhan, M., Litwack, S., Rosenbloom, D., & Keane, T. M. (2021). Public implementation of a web-based program for veterans with risky alcohol use and PTSD: A RE-AIM evaluation of VetChange. *Journal of Substance Abuse Treatment*, 122, 108242. 10.1016/j.jsat.2020.108242 33509419

[cit0011] Geretto, M., Ferrari, M., De Angelis, R., Crociata, F., Sebastiani, N., Pulliero, A., Au, W., & Izzotti, A. (2021). Occupational exposures and environmental health hazards of military personnel. *International Journal of Environmental Research and Public Health*, 18(10), 5395. 10.3390/ijerph18105395 34070145 PMC8158372

[cit0012] Glasgow, R. E., Vogt, T. M., & Boles, S. M. (1999). Evaluating the public health impact of health promotion interventions: The RE-AIM framework. *American Journal of Public Health*, 89(9), 1322–1327. 10.2105/ajph.89.9.1322 10474547 PMC1508772

[cit0013] Greenhalgh, T., Wherton, J., Papoutsi, C., Lynch, J., Hughes, G., A’Court, C., Hinder, S., Fahy, N., Procter, R., & Shaw, S. (2017). Beyond adoption: A new framework for theorizing and evaluating nonadoption, abandonment, and challenges to the scale-up, spread, and sustainability of health and care technologies. *Journal of Medical Internet Research*, 19(11), e367. 10.2196/jmir.8775 29092808 PMC5688245

[cit0014] Hu, L., & Bentler, P. M. (1999). Cutoff criteria for fit indexes in covariance structure analysis: Conventional criteria versus new alternatives. *Structural Equation Modeling*, 6(1), 1–55. 10.1080/10705519909540118

[cit0015] Kerr, N. C., Lane, S. J., Plotnikoff, R. C., & Ashby, S. (2023). The “transition” to civilian life from the perspective of former serving Australian Defence Force members. *Journal of Veterans Studies*, 9(1), 129–142. 10.21061/jvs.v9i1.407

[cit0016] Lieberman, D. A. (2012). Digital games for health behavior change: Research, design, and future directions. In S. M. Noar & N. G. Harrington (Eds.), *eHealth applications: Promising strategies for behavior change* (pp. 110–127). Routledge.

[cit0017] Livieri, G., Mangina, E., Protopapadakis, E. D., & Panayiotou, A. G. (2025). The gaps and challenges in digital health technology use as perceived by patients: A scoping review and narrative meta-synthesis. *Frontiers in Digital Health*, 7, 1474956. 10.3389/fdgth.2025.1474956 40212901 PMC11983460

[cit0018] MacLean, M. B., Van Til, L., Thompson, J. M., Sweet, J., Poirier, A., Sudom, K., & Pedlar, D. J. (2014). Postmilitary adjustment to civilian life: Potential risks and protective factors. *Physical Therapy*, 94(8), 1186–1195. 10.2522/ptj.20120107 23766397

[cit0019] Mair, J. L., Hashim, J., Thai, L., Tai, E. S., Ryan, J. C., Kowatsch, T., Müller-Riemenschneider, F., & Edney, S. M. (2025). Understanding and overcoming barriers to digital health adoption: A patient and public involvement study. *Translational Behaviour Medicine*, 15(1). 10.1093/tbm/ibaf010 PMC11959363 40167046

[cit0020] McCaslin, S. E., Becket-Davenport, C., Dinh, J. V., Lasher, B., Kim, M., Choucroun, G., & Herbst, E. (2021). Military acculturation and readjustment to the civilian context. *Psychological Trauma*, 13(6), 611–620. 10.1037/tra0000999 33507793

[cit0021] McGinty, E. E., Alegria, M., Beidas, R. S., Braithwaite, J., Kola, L., Leslie, D. L., Moise, N., Mueller, B., Pincus, H. A., Shidhaye, R., Simon, K., Singer, S. J., Stuart, E. A., & Eisenberg, M. D. (2024). The Lancet Psychiatry Commission: Transforming mental health implementation research. *Lancet Psychiatry*, 11(5), 368–396. 10.1016/s2215-0366(24)00040-338552663

[cit0022] Pedlar, D., Thompson, J. M., & Andrew Castro, C. (2019). Chapter 3 - Military-to-civilian transition theories and frameworks. In C. A. Castro & S. Dursun (Eds.), *Military veteran reintegration* (pp. 21–50). Academic Press. 10.1016/B978-0-12-815312-3.00003-6

[cit0023] Ravindran, C., Morley, S. W., Stephens, B. M., Stanley, I. H., & Reger, M. A. (2020). Association of suicide risk with transition to civilian life among US military service members. *JAMA Network Open*, 3(9), e2016261. 10.1001/jamanetworkopen.2020.16261 32915235 PMC7489860

[cit0024] Riva, G., Terruzzi, T., & Anolli, L. (2003). The use of the internet in psychological research: Comparison of online and offline questionnaires. *Cyber Psychology & Behavior*, 6(1), 73–80. 10.1089/109493103321167983 12650565

[cit0025] Romaniuk, M., Fisher, G., Kidd, C., & Batterham, P. J. (2020). Assessing psychological adjustment and cultural reintegration after military service: Development and psychometric evaluation of the post-separation Military-Civilian Adjustment and Reintegration Measure (M-CARM). *BMC Psychiatry*, 20(1), 531. 10.1186/s12888-020-02936-y 33167907 PMC7654614

[cit0026] Romaniuk, M., & Kidd, C. (2018). The psychological adjustment experience of reintegration following discharge from military service: A systematic review. *Journal of Military and Veterans Health*, 26(2), 60–73. 10.2021/33613133/JMVHVol26No2

[cit0027] Royal Commission into Defence and Veteran Suicide. (2024). *Final report (volumes 1-9)*. Commonwealth of Australia.

[cit0028] Runnals, J. J., Garovoy, N., McCutcheon, S. J., Robbins, A. T., Mann-Wrobel, M. C., & Elliott, A. (2014). Systematic review of women veterans’ mental health. *Womens Health Issues*, 24(5), 485–502. 10.1016/j.whi.2014.06.012 25213742

[cit0029] Sachdev, S., & Dixit, S. (2024). Military to civilian cultural transition experiences of retired military personnel: A systematic meta-synthesis. *Military Psychology*, 36(6), 579–592. 10.1080/08995605.2023.2237835 37490333 PMC11622583

[cit0030] Stevens, J. P. (2012). *Applied multivariate statistics for the social sciences*. Routledge.

[cit0031] Van Hooff, M., Lawrence-Wood, E., McFarlane, A., & Van Til, L. (2018). The Australian Defence Force mental health prevalence and wellbeing study. *The Medical Journal of Australia*, 209(7), 305–310. 10.5694/mja17.01152

[cit0032] Yentes, R., & Wilhelm, F. (2023). *Procedures for computing indices of careless responding*. https://cran.r-project.org/web/packages/careless/refman/careless.html

[cit0033] Zhao, Y., Summers, R., Gathara, D., & English, M. (2024). Conducting cross-cultural, multi-lingual or multi-country scale development and validation in health care research: A 10-step framework based on a scoping review. *Journal of Global Health*, 14, 04151. 10.7189/jogh.14.04151 39024643 PMC11257704

[cit0034] Zimmerman, M. (2024). The value and limitations of self-administered questionnaires in clinical practice and epidemiological studies. *World Psychiatry*, 23(2), 210–212. 10.1002/wps.21191 38727038 PMC11083863

