# Supplementary material for: Evaluating real-world uptake of the online Military to Civilian Adjustment and Reintegration Measure among former military members
Source: Aust J Psychol. 2026 May 3;78(1):2664166. doi: 10.1080/00049530.2026.2664166 (PMC13137745; doi:10.1080/00049530.2026.2664166)
Supplement: Supplemental material [file RAUP_A_2664166_SM6539.pdf]

Supplemental online material

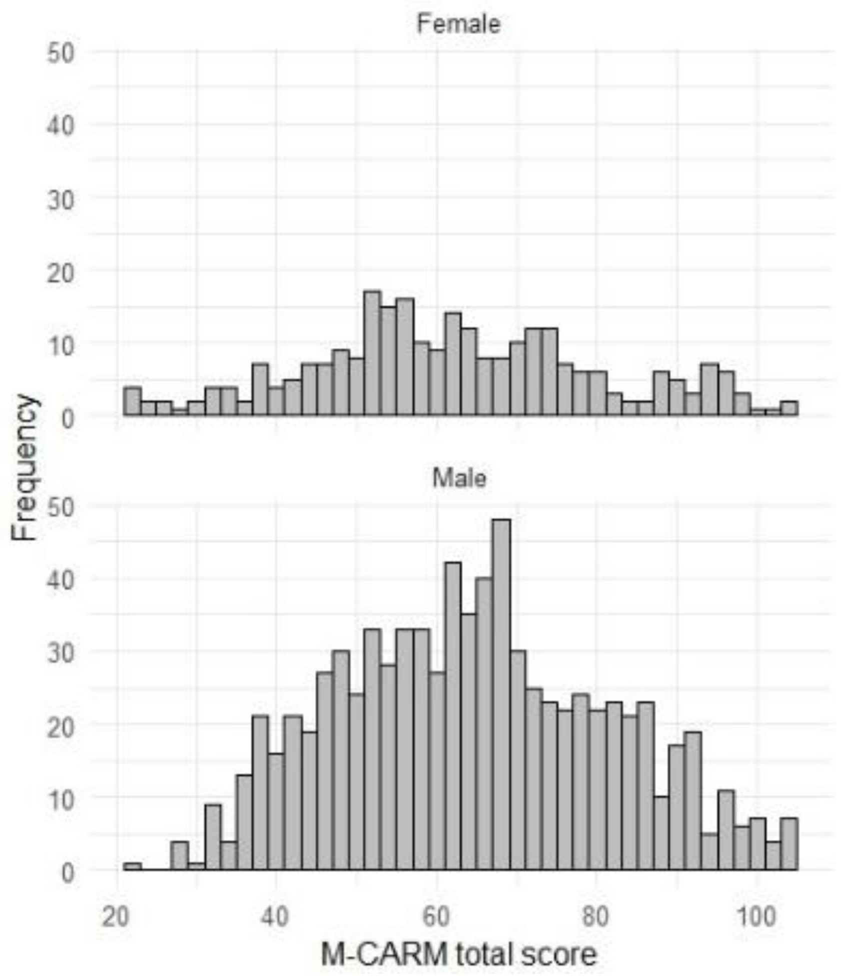

Supplementary Figure 1. M-CARM total score distribution by gender.

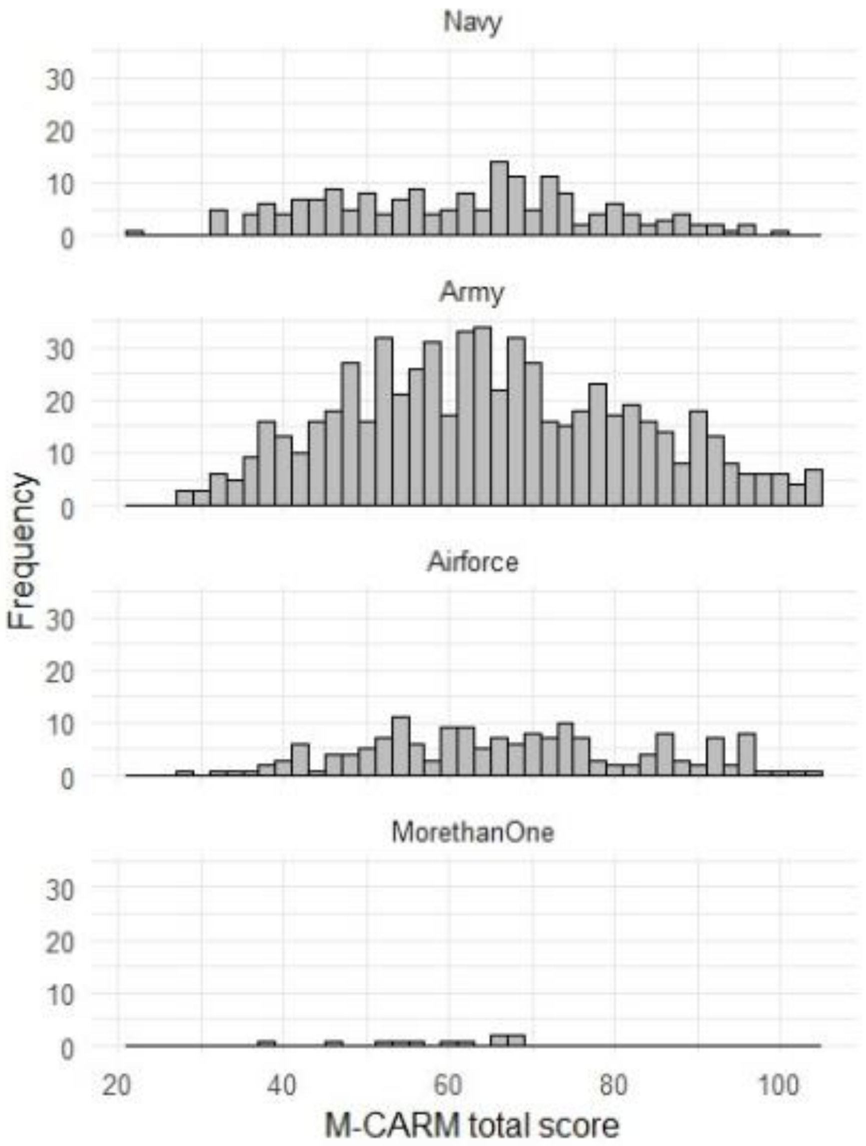

Supplementary Figure 2. M-CARM total score distribution by service type.

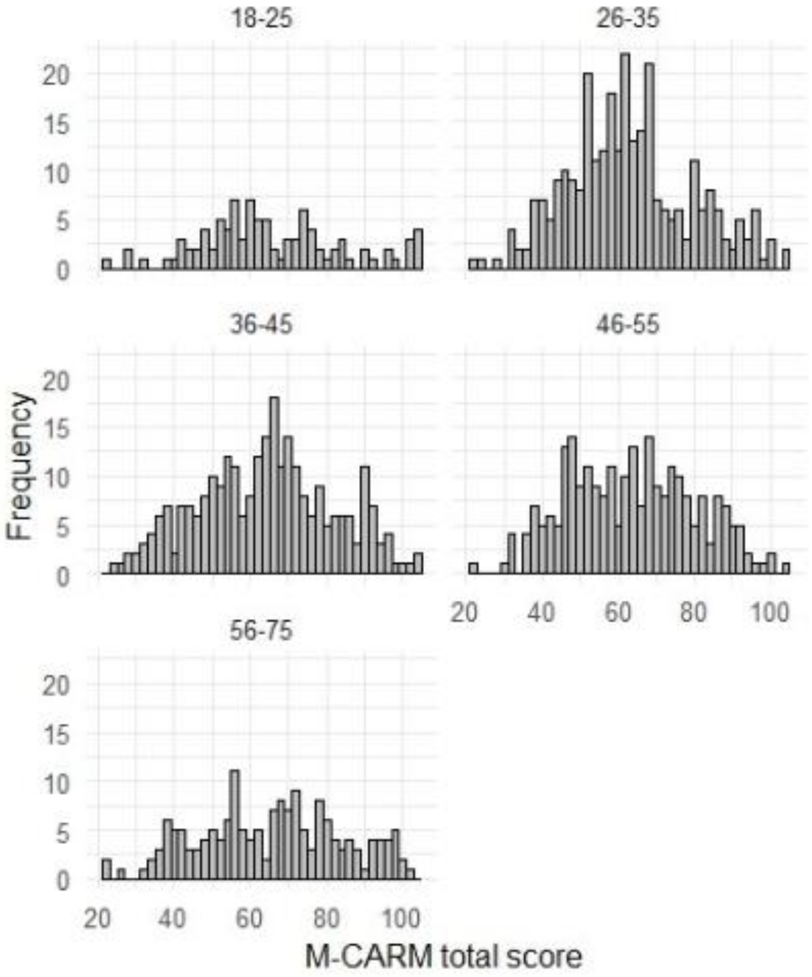

Supplementary Figure 3. M-CARM total score distribution by age group.
